# The public health consequences of mandatory return migration: a call for action

**DOI:** 10.3389/fpubh.2025.1577018

**Published:** 2025-04-30

**Authors:** Juan S. Izquierdo-Condoy, José Pablo Salazar-Aguilar, Jorge Vasconez-Gonzalez, Esteban Ortiz-Prado

**Affiliations:** ^1^One Health Research Group, Universidad de las Américas, Quito, Ecuador; ^2^Escuela de Comunicación Colectiva, Universidad Latina de Costa Rica, San Pedro, Costa Rica

**Keywords:** return migration, public health, health disparities, reintegration, migration policy

## Abstract

Migration has long shaped human societies, often generating complex social and political dynamics. In the United States, migration from Latin America represents a significant proportion of inflows, but increasingly restrictive policies have intensified hardships for migrants. Returning migrants frequently encounter systemic barriers such as limited healthcare access, economic instability, and social exclusion, all of which contribute to widening health disparities. The “healthy migrant effect” often declines as migrants face limited medical resources, reintegration difficulties, and weakened social support networks, which heighten the risk of mental health issues such as depression and PTSD. Moreover, food insecurity, poor living conditions, and exposure to violence further exacerbate physical and mental health vulnerabilities. Forced return migration magnifies these risks, leading to the marginalization of returnees both socially and economically. Addressing these challenges requires coordinated, equity-focused migration policies that integrate public health, legal, and social support systems. Sustainable, rights-based approaches are essential to promoting the long-term wellbeing of migrants and achieving broader public health goals.

## 1 Introduction

Over the past few decades, global migration flows have intensified, with a marked acceleration in the last 10 years driven by economic instability, armed conflict, and more recently, the HIV/AIDS pandemic. According to the United Nations High Commissioner for Refugees (UNHCR), a migrant is defined as an individual who voluntarily leaves their country of origin, retaining the ability to return home safely at any time (1). Additionally, return migration encompasses a range of movements back to the country of origin, including repatriation, expulsion, deportation, assisted return, and voluntary return initiated by the individual (2). Contemporary migration patterns are especially prominent in flows from Latin America to the United States and from Africa to Europe. In response, destination countries have implemented increasingly restrictive immigration policies. Among these is the recent adoption of mandatory return migration, which obliges migrants to return to their countries of origin regardless of their specific circumstances or humanitarian and health-related needs.

This phenomenon has significant implications for public health, particularly for undocumented immigrants, a group that may include individuals facing a double burden of vulnerability—a condition in which general social and legal marginalization is compounded by the presence of specific health conditions, further increasing their risk (3). These individuals often lack legal status, have limited or no access to health care, and live under the constant threat of Immigration and Customs Enforcement (ICE) raids, all of which intensify their economic insecurity and psychological distress. The intersection of migration and health thus emerges as a critical public health concern. Studies have shown that returned migrants frequently experience poorer health outcomes, largely due to restricted access to medical services, persistent economic hardship, and the stress associated with reintegration into their countries of origin (4, 5).

These implications have become increasingly evident in recent policy decisions. On January 20, 2025, President Trump signed an executive order suspending the U.S. Refugee Admission Program (USRAP) indefinitely (6). This drastic policy shift has sparked intense debate, particularly regarding its public health implications and the potential human rights violations faced by undocumented migrants (7). Despite the profound disruptions caused by mandatory return migration, its health consequences often remain underrecognized by authorities (7).

Reintegration following deportation presents a distinct set of challenges that further undermine migrant wellbeing. Deported migrants are not merely leaving a country, they are being forcibly removed from established lives, where they have built homes, raised children, and integrated into society. Research indicates that return migrants face substantial barriers to healthcare access, further worsened by social exclusion, economic instability, and the psychological trauma of forced displacement. These factors compound their vulnerability, making reintegration into their home countries particularly challenging (8–10).

Furthermore, disparities in income and social support systems contribute to unequal health outcomes between returning migrants and urban populations—a group often used as a benchmark due to their relatively greater access to healthcare services and social infrastructure. These disparities frequently heighten returnees' vulnerability to both physical and mental health conditions (11–13). The lack of reintegration programs—combined with economic instability and limited access to healthcare—places returnees at a marked disadvantage, exacerbating pre-existing inequities and perpetuating cycles of poverty and social exclusion (14). Notable examples of reintegration initiatives include Mexico's 3 × 1 Program for Migrants and Ecuador's Emergency Assistance Plan for nationals returning from the United States (15, 16).

To better understand these dynamics, the findings from the literature were synthesized to identify key trends, gaps in existing research, and to highlight the long-term effects of deportation and repatriation under the current U.S. immigration policies.

## 2 Methodology

This perspective study evaluates the public health impacts of mandatory return migration, specifically following the implementation of immigration policies under President Trump's administration. A comprehensive literature review was conducted to identify relevant studies assessing the health outcomes of return migrants. The review was carried out using the PubMed, Scopus, and Google Schoolar databases, with the following search terms: (“Return Migrant” OR “Return Migration”) AND (“public health impact” OR “health risk” OR “health outcomes”). Out of 150 sources initially screened, 12 peer-reviewed articles were selected for inclusion in this analysis.

The data were analyzed across key areas, including the physical health risks of mandatory return migration, focusing on limited healthcare access and the worsening of pre-existing conditions, such as physical health problems, health issues, psychological and mental health problems, violence and harm based on gender or sexual orientation (17). The role of social support and its impact on the reintegration and wellbeing of return migrants was also explored. Additionally, the review assessed how lifestyle factors—such as diet, physical activity, and socioeconomic status—affect the health outcomes of returnees, with a particular emphasis on migration's influence. Psychological challenges, including stress, anxiety, and depression resulting from forced displacement and reintegration, were examined. Lastly, the review addressed the ethical and policy implications of mandatory return migration, evaluating the effectiveness of related health programs in both sending and receiving countries.

## 3 Public health impacts of mandatory return migration

Mandatory return migration can have profound public health implications, particularly for vulnerable populations such as migrant workers. The intersection of health outcomes and social support systems plays a crucial role in shaping the wellbeing of these individuals, influencing both short- and long-term reintegration challenges (18).

### 3.1 Health risks associated with mandatory return migration

The *healthy migrant effect* suggests that migrants initially arrive in their host countries in better health than the local population (19). However, this advantage is often short-lived due to various challenges encountered abroad, including poor working conditions, limited access to healthcare, and social discrimination (17, 20, 21). Upon returning to their home countries, many migrants face additional barriers to reintegration, including persistent health issues, inadequate medical resources, and socioeconomic instability. Furthermore, systemic exclusion and marginalization exacerbate these health risks, as observed in countries such as South Africa, where returning migrants face structural barriers to healthcare. Similar challenges may currently affect migrants reintegrating into regions with restricted healthcare access (8).

### 3.2 Social support and quality of life

Social support is a critical determinant of health and wellbeing, particularly for individuals experiencing forced reintegration under inhumane conditions, such as transport under forced starvation or the use of handcuffs despite individuals not being criminals. Strong social networks can mitigate stress, improve mental health, and facilitate smoother reintegration (22–24). However, social support levels vary. While some studies indicate that migrant workers report higher social support than urban workers, these findings are influenced by factors such as age, economic status, and access to reintegration programs. In the absence of adequate social safety nets, returning migrants are more likely to experience isolation, economic hardship, and deteriorating health outcomes (14, 25).

### 3.3 Lifestyle factors and health outcomes

A healthy lifestyle—characterized by balanced nutrition, regular physical activity, and avoidance of harmful behaviors such as smoking and excessive alcohol consumption—is closely associated with better quality of life. However, returning migrants often face adverse conditions that hinder their ability to maintain a healthy lifestyle, including food insecurity and unstable employment. Workplace- and community-based interventions could promote healthier behaviors, serving as effective public health strategies to mitigate these risks (25). The International Organization for Migration established a series of recommendations, such as involving migrants and local communities in all phases of activities to increase their participation, designing and incorporating activities that create a welcoming environment for people to gather, interact, and forge social bonds (26). Riza et al., in their systematic review, evaluated best practices in interventions, including community-based mental health services, a cardiovascular disease prevention program that is linguistically and culturally sensitive, and a health promotion initiative that integrates positive development principles for minority youth (27).

Undocumented migrants, in particular, face significant barriers to healthcare access due to their immigration status, which often renders them ineligible for public health insurance programs and unable to obtain private health insurance. As a result, they are less likely to have a regular healthcare provider, seek preventive services, or access specialized care, all of which contribute to poorer health outcomes (28). Moreover, the fear of deportation due to restrictive policies further deters migrants from seeking healthcare or following healthcare provider recommendations (29, 30).

Migrants are also vulnerable to violent victimization, with limited recourse for seeking help due to their fear of interacting with law enforcement. Common forms of victimization include verbal and physical assault, mistreatment by immigration and law enforcement officers, and racial or linguistic profiling, especially in workplace, healthcare, and immigration-related contexts (28).

Additionally, migrants may face heightened risks for certain health conditions, including HIV, especially among those with prior traumatic experiences or who belong to sexual minority groups. Contributing factors include discrimination, unstable housing, exposure to high-risk sexual partners, and limited access to healthcare services (28). For undocumented women, fear of deportation may lead to remaining in abusive relationships, further compounding health risks, including the potential for HIV transmission (31).

### 3.4 Mental health considerations

The mental health challenges faced by returning migrants are significant and frequently overlooked. Many migrants experience traumatic events throughout the migration process, including departure, life in foreign countries, and forced return. Prolonged uncertainty, involuntary repatriation, and socioeconomic instability contribute to a higher prevalence of anxiety, depression, and post-traumatic stress disorder (PTSD) among returnees (25). Over 75% of Latin American migrants to the U.S. report experiencing trauma, which includes pre-migration factors such as war, terrorism, political persecution, and natural disasters; migration-related exposures like robbery, kidnapping, rape, extortion, assault, and dehydration; and post-migration factors, including neighborhood and domestic violence (32).

Reintegration into communities of origin is often challenging due to stigma, disrupted social ties, and limited access to mental health services. Addressing these concerns requires a multidisciplinary approach, incorporating culturally competent mental health support, community engagement, and policy frameworks that prioritize psychological wellbeing (25). A relevant example is the López Obrador administration's response to migration, which emphasizes economic and social investment in countries of origin to combat poverty and violence, while also ensuring humanitarian treatment of migrants in Mexico. This approach includes providing an unprecedented number of humanitarian visas to victims or witnesses of serious crimes, unaccompanied minors, and individuals seeking refugee status (33, 34).

Humanitarian visas can also be granted in specific circumstances, such as when individuals face a risk to their health or life, are vulnerable to deportation, or have particular needs (e.g., pregnancy, old age, disability, indigeneity) that make deportation difficult or impossible. In cases of extreme violence or natural disasters, individuals may also be granted humanitarian visas to ensure their safety and wellbeing (34).

### 3.5 Ethical and policy considerations

Mandatory return migration policies raise significant ethical concerns. The forced repatriation of individuals without adequate support mechanisms exposes them to social marginalization and heightened vulnerability. These policies often undermine fundamental human rights and disproportionately affect already disadvantaged populations (8, 35). Governments have a duty to protect the wellbeing of all individuals within their jurisdiction, including returnees. Policies that fail to account for the health and social needs of returning migrants not only exacerbate individual suffering but also contribute to broader public health crises (36).

## 4 Discussion

The health consequences of forced return migration under President Donald Trump's immigration policies extend beyond individual migrant experiences, reflecting broader systemic barriers. The deficiencies and variations in reception systems for return migrants, who are at significant risk of vulnerability and exclusion, are a global concern (37). Addressing these challenges necessitates a multidisciplinary, evidence-based approach that prioritizes equity in access to comprehensive healthcare services and social reintegration. Such as integrating public health, social work, and legal services to deliver coordinated care tailored to the complex needs of returnees. For example, joint initiatives between health clinics, legal aid organizations, and local governments can ensure migrants receive medical treatment, housing assistance, and legal documentation support (38).

Migration policies inherently influence public health outcomes and should be designed to protect the wellbeing of returning migrants rather than exacerbate their vulnerabilities. In the absence of proactive, health-focused interventions—such as mobile health units, culturally tailored mental health programs, and preventive screening campaigns—forced return migration will perpetuate existing health disparities, imposing additional burdens on both returnees and their communities of origin (39).

Social work is uniquely positioned to address these challenges, given its focus on intervention in situations of social need and promoting the protection of human rights (37). New policies must prioritize the prevention of health issues among migrants, particularly those stemming from migration as a social determinant of health. This includes expanding eligibility for public healthcare services to undocumented returnees or removing legal barriers to access for essential services (40).

A novel approach is needed to address the health needs of specific migrant populations or individual illnesses—for example, creating community-based health networks that focus on returning women exposed to gender-based violence or designing mental health interventions specifically for unaccompanied minors (41). Traditional administrative frameworks may not effectively meet the needs of an increasingly diverse and globalized population.

Limited access to healthcare, economic instability, and social exclusion pose substantial barriers to reintegration, exacerbating both physical and mental health issues. Governments and international organizations must acknowledge the intrinsic link between migration and health, developing coordinated policies that integrate healthcare support with reintegration programs—such as reintegration case management systems that include psychosocial support, employment placement, and continued medical care post-deportation (27).

Implementing migration policies that center on health and human dignity—such as eliminating detention-based deportation procedures or prioritizing health assessments prior to return— is not only an ethical responsibility but also a global public health imperative, especially in the Global South, where fragile health systems are often ill-equipped to support returning migrants (42).

Sustainable solutions must address both immediate healthcare needs and the long-term social determinants of health. Examples include investing in cross-border healthcare access agreements, establishing returnee health monitoring systems, and supporting livelihood programs that promote economic independence post-return (43). International cooperation is essential to achieving objectives such as those outlined in the Global Compact for Safe, Orderly, and Regular Migration and the Sustainable Development Goals, including universal health coverage (42).

## 5 Conclusions

Mandatory return migration policies pose significant public health risks, particularly for vulnerable populations facing limited access to healthcare, economic hardship, and social exclusion. These conditions heighten the risk of both physical and mental health issues. Reintegration without adequate support often deepens existing inequalities. Addressing these challenges requires coordinated, equity-driven policies that integrate healthcare, social services, and legal assistance. Policymakers must prioritize the health and dignity of returnees, especially in low-resource settings. Sustainable, rights-based strategies aligned with international frameworks are essential to promote long-term wellbeing and mitigate the public health impact of forced return migration.

## Data Availability

The original contributions presented in the study are included in the article/supplementary material, further inquiries can be directed to the corresponding author.
